# MiRNA-126 expression inhibits IL-23R mediated TNF-α or IFN-γ production in fibroblast-like synoviocytes in a mice model of collagen-induced rheumatoid arthritis

**DOI:** 10.1007/s10495-018-1474-7

**Published:** 2018-08-24

**Authors:** Jie Gao, Ruina Kong, Xiaoli Zhou, Lianmei Ji, Ju Zhang, Dongbao Zhao

**Affiliations:** 1 Department of Rheumatology and Immunology, Changhai Hospital, Second Military Medical University, No. 168 Changhai Road, Shanghai, 200433 China; 2 Department of Pathology, Changzhou Second People’s Hospital, Changzhou, China

**Keywords:** Rheumatoid arthritis, Fibroblast-like synoviocytes, MiRNA-126, IL-23R

## Abstract

Both miR-126 and IL-23R affect rheumatoid arthritis (RA) procession. This study aimed to investigate the association of miR-126 and IL-23R and the possible modulation of miR-126 to RA pathogenesis. Serum, synovial tissue and synovial fluid were collected from patients with RA, and expression of miR-126, IL-23R, TNF-α and IFN-γ were detected. Fibroblast-like synoviocytes (FLS) was established using a collagen-induced arthritis mice model. The expression of miR-126 was manual intervened using pro-miR-126 and anti-miR-126 encoding lentivirus plasmids, or miR-126 agonists and corresponding negative controls. MiR-126 expression was inhibited in RA patients when compared with controls (P < 0.05). TNF-α and IFN-γ production and IL-23R expression were significantly upregulated in RA patients when compared to controls (P < 0.05). In pro-miR-126 treated FLS cells, the administration of pro-miR-126 plasmids upregulated miR-126, but inhibited IL-23R, TNF-α and IFN-γ expression or production. Moreover, the miR-126 agonist reversed the effects of the anti-miR-126 plasmid on FLS. These results revealed that miR-126 negative regulated the expression of IL-23R, TNF-α and IFN-γ. These results suggest the key impact of miR-126 on RA procession. Moreover, pro-miR-126 might be explored to be a potential therapy for RA.

## Introduction

Rheumatoid arthritis (RA) is a systematic inflammatory disease that relates to the overexpression of pro-inflammatory cytokines [1, 2]. The abnormal upregulation of cytokines including Th17-type TNF-α or IFN-γ is prevalent in RA patients, and RA patients could be characterized by increased TNF-α [3]. TNF-α stimulates proinflammatory factors and promotes the activity of osteoclasts, thereby resulting in RA or other inflammatory processions.

The association of interleukin-23 (IL-23) or IL-23 receptor (IL-23R) and inflammatory diseases has been prevalently reported [4–6]. It was reported that IL-23 mediates the generation, stability and proliferation of Th17 cells, as well as the production of IL-17 [7, 8]. The regulation of IL-23–Th17 axis activity determines the onset of the inflammatory disease [9]. Moreover, polymorphisms in IL-23R and IL-23 are prevalent in RA, and are associated with the overproduction of IL-17, TNF-α, or IFN-γ [10]. Anti-IL-23 could be used as therapy for inhibiting the inflammatory pathway [11].

The genetic analysis for microRNA (miRNA) in RA revealed that miRNAs are largely related to RA procession [12–16]. MiRNAs are involved in RA via mediating the osteoclast/osteoblast differentiation, generation, stability and proliferation of Th17 and Treg cells, which are agents that are resistant to rituximab [14–18]. For example, miR-21 has been proven to promote the proliferation of fibroblast-like synoviocytes (FLS) in collagen-induced RA via the NF-κB pathway [18]. Furthermore, miR-126 has been reported to affect the proliferation and apoptosis of RA synovial fibroblasts by targeting P1K3R2 via the P13K–AKT pathway [19]. Moreover, abnormalities in miR-126 expression have been reported to be correlated to DNA hypomethylation in T-cells of RA patients [20, 21].

We investigated IL-23R as a target of miR-126. In order to determine the association of miR-126 and IL-23R and the possible modulation of miR-126 to RA pathogenesis, we detected the expression of miR-126, IL-23R and cytokines TNF-α and IFN-γ in RA patients and FLS in vitro. The expression of miR-126 was manually intervened by pro-miR-126 and anti-miR-126 plasmids or agonists to explore the association among those factors above. This study would provide new insights into miR-126 related RA pathogenesis.

## Materials and methods

### RA patients, sample collection and processing

All experiment protocols were approved by the Ethics Committee of Changhai Hospital. Written consent was obtained from participants. A total of 61 RA patients (average age: 55.4 ± 3.5 years old; 23 male and 38 female patients) were included in the present study. All patients fulfilled the American College of Rheumatology criteria for classification of RA [22]. Peripheral blood and synovial fluid were sampled for ELISA measurement, and synovial tissue were collected, snap-frozen and stored at − 80 °C for protein, mRNA and miRNA detection.

### Establishment of RA model

All protocols of animal experiments were reviewed and approved by the Institutional Animal Care and Use Committee at the Changhai Hospital, Second Military Medical University. The Lewis rat (180 ± 20 g, 45 days old) collagen-induced arthritis model was established using a previously reported method [18]. Twenty rats (20 male and 20 female) were randomly divided into two groups after being acclimated for 8 days prior to type II collagen injection (n = 10, each group): sham control group and RA group. Rats in the RA group received an injection at the base of the tail and back of 0.5 mL of complete Freund’s adjuvant (CFA; Sigma-Aldrich, St Louis, MO, USA) supplemented with 4 mg/mL of bovine type II collagen (CII; Elastin Products, Owensville, Missouri) on day zero and seven. Rats in sham control group received an injection of normal saline. Rats were housed in a 12 h-light/12 h-dark environment with 30–70% relative humidity at 67–76 °F, and were given free access to food and water. Finally, all animals were decapitated; and the synovial tissue was stripped, rinsed, sterilized, snap–frozen, and stored at − 80 °C.

### FLS isolation, culture and transfection

The isolation and culture of FLS were performed according to a previous method [18]. Synovial tissues isolated from the RA model were cut into 1-mm^3^ blocks in DMEM (Sigma), and were evenly sprayed onto the bottom of the flasks with DMEM containing 10% FCS (Sigma). The flasks were incubated at 37 °C with 5% CO_2_, and the medium was replaced every 2 days for passaging. Cells at passages 3–4 were used for the present study. For miR-126 inhibition or activation, 1 × 10^4^ cells were treated with lentiviruses encoding pro-miR-126, anti-miR-126, or control (empty plasmid), miR-126 agonist and negative control (50 ng/mL) using Lipofectamine 2000 (Invitrogen, Carlsbad, CA) for 24 h. The pro-miR-126 and anti-miR-126 encoding lentiviruses plasmids were purchased from Shanghai Sangon Biotechnology Co., Ltd. (Shanghai, China).

#### RT-PCR

Total RNA was dissociated from tissues using Trizol reagent (Invitrogen, USA) and the first strand cDNA was reverse-transcribed using a cDNA synthesis kit (BioRad, Hercules, CA, USA). Reactions were conducted on an ABI7500 Real-Time PCR system (Applied Biosystems/Life Technologies, Carlsbad, CA, USA). Then, 25 µL of reaction mixture was amplified according the following steps: initial desaturation at 95 °C for 5 min, 35 cycles of denaturation at 95 °C for 30 s, annealing at 60 °C for 30 s, and extension at 72 °C for 10 s. Internal reference gene GAPDH was employed. The primer pairs used for miRNA and mRNA amplification are synthesized by Shanghai Sangon Biotechnology Co., Ltd. (Shanghai, China) as follows: miR-126, forward, 5′-GCUCGUACCGUGAGUAAU-3′; reverse, 5′-CAGTGCAGGGTCCGAGGT-3′; IL-23R, forward, 5′-AAGAAGACAGCACAGCCAG-3′, reverse, 5′-CAAGAGTTCAGCCATCCTC-3′; IFN-γ, forward, 5′-TCAAGTGGCATAGATGTGGAAGAA-3′, reverse, 5′-TGGCTCTGCAGGATTTTCATG-3′; TNF-α, forward, 5′-TAGCCCACGTCGTAGCA-3′, reverse, 5′-GGGGTCAGAGTAAAGGGGTC-3′; GAPDH forward, 5′-CACCCACTCCTCCACCTTTG-3′, reverse, 5′-CCACCAC-CCTGTTGCTGTAG-3′. Each reaction was performed in triplicate. The statistical data were expressed as mean ± standard deviation (SD) (bar).

### Western blotting analysis

Isolated tissues or cells were lysed, and the protein concentration of the lysates was measured (Pierce BCA protein assay kit, Rockford, IL, USA). An equal amount of 35 µg of lysate was separated by 10% SDS–PAGE and transferred onto PVDF membranes (Millipore Corp., Carrigtwohill, Ireland). Then, this was blocked by BSA (5%, Sigma) and incubated with primary antibody against IL-23R (dilution at 1:1000; Abcam, Cambridge, MA) and GAPDH (dilution at 1:1000; Epitomics, Burlingame, CA, USA) at 4 °C for 24 h. After washing, the membranes were incubated with HRP-labeled secondary antibodies (dilution at 1:2000) at room temperature for 1 h. The signal intensities were quantified.

### ELISA assay

The detection of human and rat serum, as well as cellular TNF-α and IFN-γ content, were performed using ELISA kits (Elabscience, Wuhan, China).

### Statistical analysis

Statistical analysis was performed using SPSS 16.0 statistical package. Quantitative data were expressed as mean ± SD. Student’s *t* test or ANOVA was used for differences analysis. A P value < 0.05 was considered statistically significant.

## Results

### MiR-126, IL-23R and cytokines were upregulated in RA

**Fig. 1:**
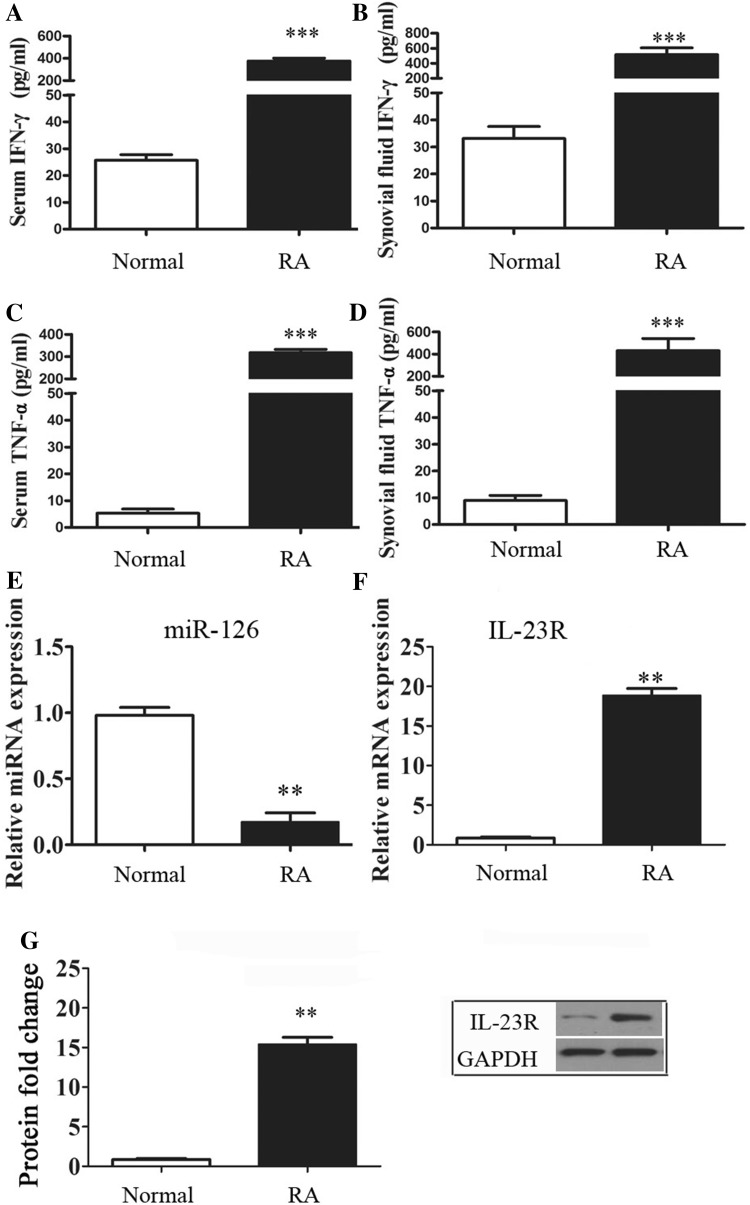
Elevated miRNA, IL-23R and cytokine levels were detected in RA patients. **a**–**d** IFN-γ and TNF-α serum and synovial fluid content were detected using an ELISA kit. **e, f** The mRNA expression of miR-126 and IL-23R were detected using RT-PCR methods. **g** Western blot was employed to detect the protein expression of IL-23R. *, ** and *** indicates significant levels at P < 0.05, 0.01, and 0.001 versus NCs, respectively

The upregulation of cytokines IFN-γ and TNF-α in serum and synovial fluid were detected in RA patients and compared to normal controls (NCs) (P < 0.001, Fig. 1a–d). Data revealed that miR-126 was inhibited in RA patients (P < 0.01, Fig. 1e). In addition, IL-23R mRNA and protein expression significantly increased in RA patients, compared to controls (P < 0.05, Fig. 1f, g). Therefore, there might be a negative relationship between miR-126 and IL-23R and cytokines in RA.

### MiR-126 inhibited the expression of IL-23R and cytokines

**Fig. 2:**
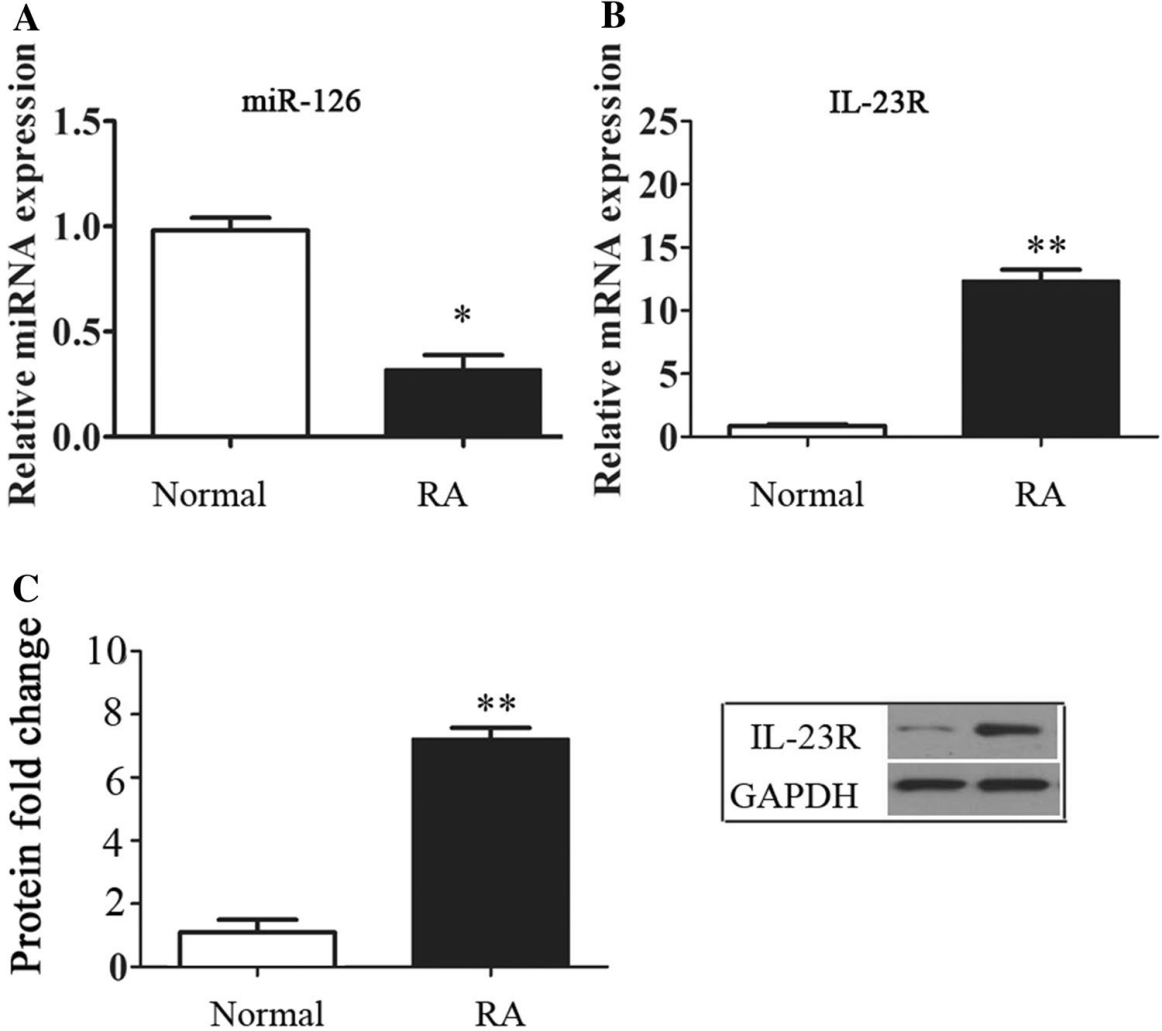
Establishment of FLS. **a**–**d** The miRNA and mRNA expression was detected by RT-PCR. **c** Represents the protein fold change of IL-23R to GAPDH. * and ** indicates significant levels at P < 0.05, and 0.01 versus NCs, respectively

**Fig. 3:**
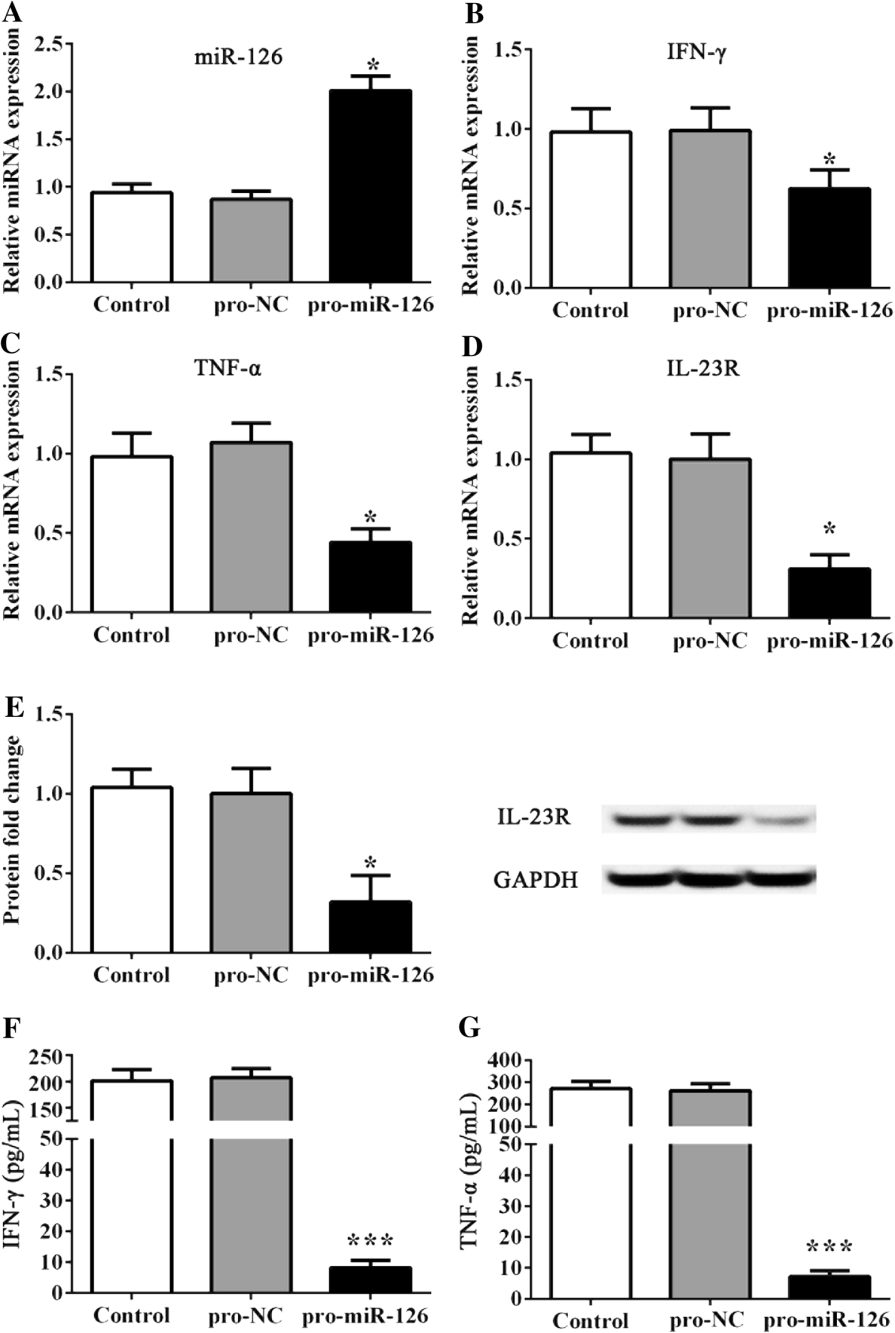
Effect of pro-miR-126 administration on FLS. **a**–**d** The miRNA and mRNA expression was detected by RT-PCR. **e** Represents the protein fold change of IL-23R to GAPDH. **f, g** IFN-γ and TNF-α cellular medium content was detected by ELISA. * and *** indicates significant levels at P < 0.05, and 0.001 versus controls or NCs, respectively

In the in vivo RA model, the inhibition of miR-126 and elevation of IL-23R in synovial tissues were confirmed (Fig. 2a–c). Then, FLS was established and treated with pro-miR-126 and anti-miR-126 lentivirus plasmids. Data revealed that the addition pro-miR-126 upregulated the expression of miR-126 (P < 0.05, Fig. 3a), and significantly inhibited the expression of IFN-γ, TNF-α and IL-23R versus controls (P < 0.05, Fig. 3b–e). In addition, the content of medium IFN-γ and TNF-α was significantly reduced by pro-miR-126 treatment (P < 0.001, Fig. 3f, g).

### MiR-126 silencing promoted the expression of IL-23R and cytokines

**Fig. 4:**
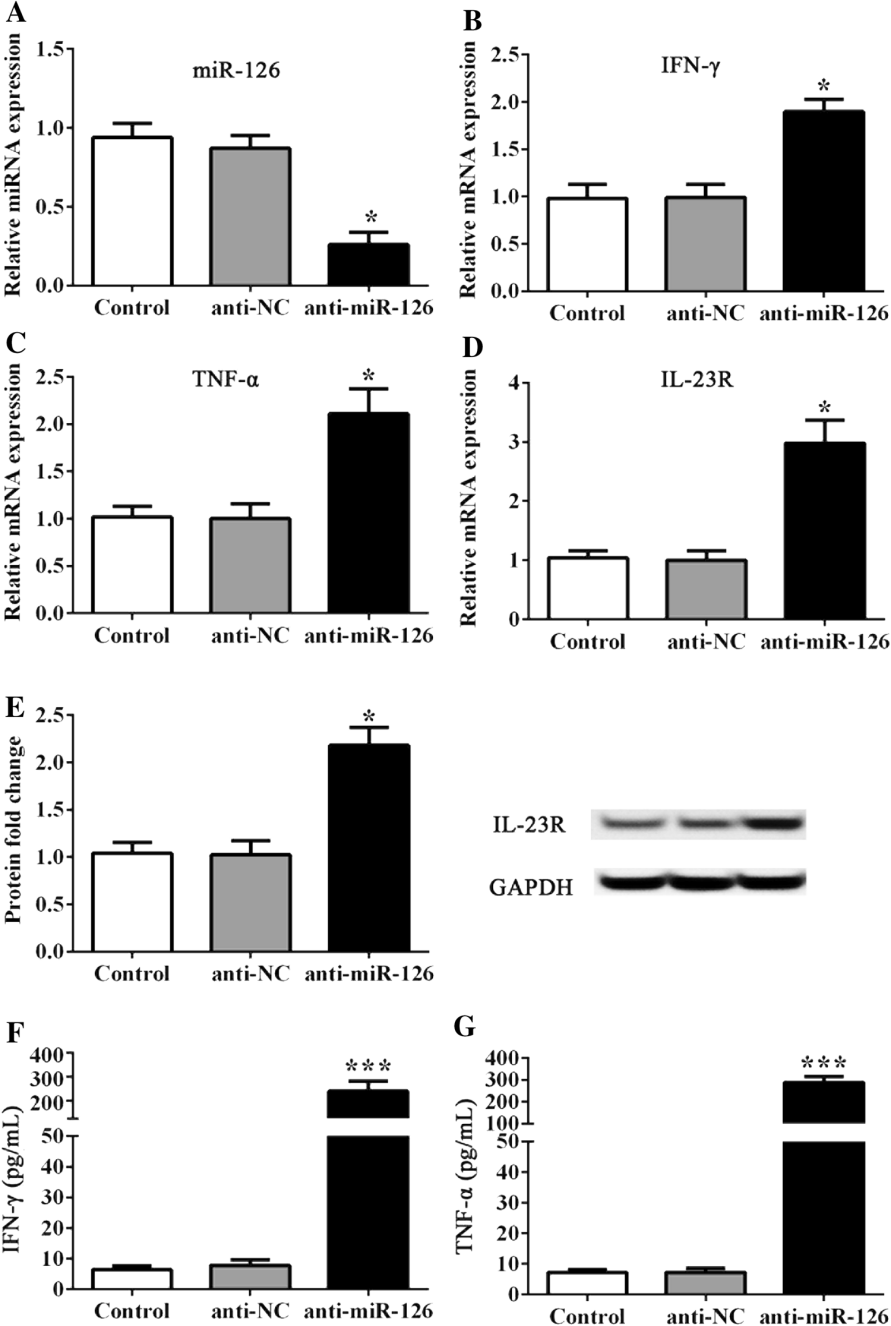
Effect of anti-miR-126 administration on FLS. **a**–**d** The miRNA and mRNA expression was detected by RT-PCR. **e** Represents the protein fold change of IL-23R to GAPDH. **f, g** IFN-γ and TNF-α cellular medium content was detected by ELISA. * and *** indicates significant levels at P < 0.05, and 0.001 versus controls or NCs, respectively

**Fig. 5:**
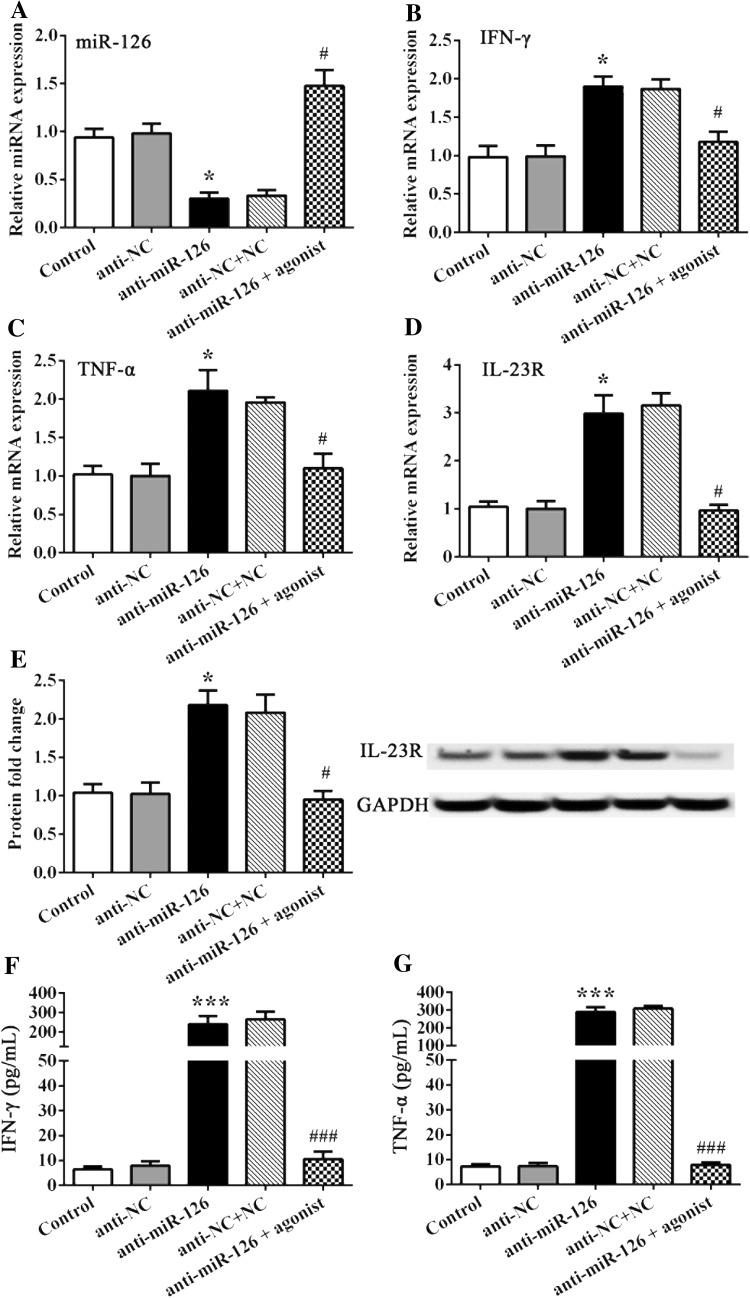
The MiR-126 agonist impedes the effect of anti-miR-126 on FLS. **a**–**d** The miRNA and mRNA expression was detected by RT-PCR. **e** Represents the protein fold change of IL-23R to GAPDH. **f, g** IFN-γ and TNF-α cellular medium content was detected by ELISA. * and *** indicates significant levels at P < 0.05, and 0.001 versus controls or NCs, respectively. ^#^ and ^###^ indicates significant levels at P < 0.05, and 0.001 versus cells treated with anti-miR-126 plasmids, respectively

On the contrary, FLS was treated with anti-miR-126 plasmids, and the inverse results of those factors above were determined. FLS treated with anti-miR-126 resulted in the suppressed expression of miR-126 (P < 0.05, Fig. 4a), while IL-23R and cytokines IFN-γ and TNF-α were significantly upregulated, when compared to controls (P < 0.05, Fig. 4b–g). However, the additional administration of the miR-126 agonist impeded the effect of anti-miR-126 on miR-126, as well as on IL-23R and IFN-γ, or TNF-α cytokines (Fig. 5b–g). All data revealed that miR-126 negatively regulated the expression of IL-23R, as well as the expression of cytokines including IFN-γ and TNF-α, in FLS in vitro.

## Discussion

The present study suggests that miR-126 modulated the expression of IL-23R and the content or extraction of cytokines of IFN-γ and TNF-α in FLS in vitro. There was a negative relationship between the expression of miR-126 and the expression of IL-23R or the content of IFN-γ and TNF-α.

The abnormal upregulation of cytokines including TNF-α or IFN-γ is prevalent in RA patients. Hence, RA patients could be characterized by increased TNF-α levels [3]. Cytokine inhibitors, including anti-TNF-α therapy, have been once accepted to be the therapy of choice for RA patients, which is presently being applied in clinic [23]. In the present study, we found that the expression of miR-126 could negatively modulate the cytokine content of TNF-α and IFN-γ. Furthermore, the inhibition of miR-126 and the overproduction of TNF-α and IFN-γ were detected in RA patients. On the contrary, pro-miR126 treatment reduced the production of TNF-α and IFN-γ in FLS in vitro, suggesting that the overexpression of miR-126 contributed to TNF-α and IFN-γ inhibition, and might be used as a therapy for RA.

In addition, the association of IL-23R or IL-23 and inflammatory diseases, and the fact that IL-23 could be used as a therapy for inhibiting the inflammatory pathway, demonstrates the close association between IL-23R and RA [4, 5, 11, 24]. The regulation of IL-23–Th17 axis activity determines the onset of the inflammatory disease [9]. IL-23 mediates the stability and proliferation of Th17 cells, and induces the production of cytokines including IL-17 and IFN-γ, which is extracted by Th17 cells [25]. In the present study, we demonstrated that the expression of IL-23r was consistently matched with the contents of TNF-α and IFN-γ in RA patients in vivo or FLS in vitro. These results reveal that IL-23R might be the key for miR-126 modulation in the RA process.

The study also has some limitations. First, polymorphisms in IL-23R and IL-23 are prevalent in RA, and are associated with the overproduction of IL-17, TNF-α, or IFN-γ in RA [10]. However, we did not study the polymorphisms of IL-23R in RA and its relationship with TNF-α and IFN-γ production. Moreover, the IL-23R/TNF-α/IFN-γ pathway and the target relationship between miR-126 and IL-23R were not explored in the present study. Studies showed that miR-126 could affect the proliferation and apoptosis of RA synovial fibroblasts by targeting P1K3R2 via the P13K–AKT pathway, however in this study target for miR-126 needs more further investigations to confirm. Despite these limitations, the results of the present study could prove the negative relationship between miR-126 and IL-23R, TNF-α, or IFN-γ, and the therapeutic potential of miR-126 for RA.

## Conclusion

We demonstrated that the downregulation of miR-126 was negatively associated with the expression of IL-23R and the contents of TNF-α and IFN-γ in RA patients. Moreover, manually intervened miR-126 overexpression inhibited the expression of IL-23R, which was accompanied by decreased TNF-α and IFN-γ content in FLS in vitro. These results suggest the key impact of miR-126 on RA procession and the therapeutic potential of miR-126 for RA. However, more experiments should focus on related mechanisms, in order to explore the association of miR-126 and RA pathogenesis and therapy.
